# Introductory Lectures. No. 2

**Published:** 1848-04

**Authors:** 


### BUFFALO MEDICAL JOURNAL

AND

### MONTHLY REVIEW.

VOL. 3. APRIL, 1848. NO. 11.

### ORIGINAL COMMUNICATIONS.

ART, I.—*Introductory Lectures.* *No.* 2.—In continuation of the plan commenced in our last No., we proceed to notice, and present to our readers extracts from the remainder of the introductory addresses with which we have been favored.

Leaving Philadelphia, we must pass by Baltimore, the city of New-York, and Albany. If any of the introductory discourses delivered at the institutions in these cities have been published, we have been overlooked in their distribution. So, also, Pittsfield, Woodstock, Hanover and Castleton are not represented in our budget. From the Medical Department of Harvard University, Boston, Mass., we have to acknowledge a copy of an introductory by Oliver Wendell Holmes, M. D., lately appointed to the chair of Anatomy and Physiology in that school.

Dr. Holmes is favorably known as author of some valuable communications on medical subjects. He enjoys greater celebrity, however, in the walks of polite literature. He furnishes an illustration of the compatibility of pursuits so antagonistical, apparently, as physic and poetry, having cultivated the latter with a degree of success which might well satisfy a reasonable ambition. But the annals of medicine are not without examples in which the characters of poet and physician have been united, and preserved, without deterioration of either character; and Apollo, if our mythological knowledge serves us, was the Divinity of the lyre, as well as of medicine. The lecture, as a literary effort, takes precedence of all which we have received; but, as regards matter and tone, we must say, it seems to our prosaic apprehension to be open to criticism. Having said thus much we are bound to adduce some of the portions of the lecture to which our remarks have reference.

Having alluded in terms of approbation to the organization of the National Medical Association, the lecturer speaks of its objects, &c., in this wise:—

"If the feeling in which this originated were truly stated, it would be found that it was a result of the institution of *inferior medical schools*, situated in the *wrong places*, and managed by the *wrong men*, and the consequent cheapening and *vulgarizing* of education, until the degree of doctor of medicine has, in *some parts* of our country, ceased to be an evidence of a decent amount of knowledge on the part of those who possess it. The honorable and thorough bred practitioner found himself shouldered by ignorant novices claiming to be his equals, who had never devoted half the allotted period of pupilage to study, who had never touched a scalpel, who had never *seen* a hospital, and in the face of an easy public, apt to take men at their own valuation, and having no proper means of discriminating between them.* Such, I believe, was the original mainspring of the movement, but other and most useful designs have been superadded to the principal one of raising the degenerated standard of education. Among them are the thorough union and organization of the profession, the establishment of an elevated code of ethics, and the pursuit of various inquiries in concert by physicians of different sections of the country.

There are two great objects, then, aimed at by the National Medical Association: one may properly be called ***Reform***; it implies the previous existence of abuses, and casts a reproach upon all to whom its measures apply, for doing some wrong, or neglecting some duty. The other is *improvement*, and this it may be presumed every body and association in the country, like every individual person, is capable of, and will find ample room for, if it is urged upon them. Now, I believe I may say with confidence, that

---

* It is a little remarkable that on the morning after these words were written, I received a letter from one of the great states of the distant West, containing the following sentences, "We have several miserable apologies for medical schools in this state, which by cheapening the rates of instruction induce many young men of limited means to attend their lectures. The consequence is that we have many among us who flourish an M. D., and yet are ignorant of the elementary principles of Medical Science."

neither this community nor this institution are obnoxous to any charge that calls for the agency of *reform* as that word is commonly intended. We *reform* the victims of habitual intoxication, but if we induce a strictly temperate person to become more severely abstinent, we may call it improvement if we will, but not reformation. Our medical community was already thoroughly organized; an ethical code, in most respects identical with that adopted by the National Convention, had been in action here for a long series of years; a general state of harmony and an enlightened public sentiment were already prevalent. In this University there was a full complement of instructors; the first of all requisites, clinical teaching, was thoroughly attended to, and the full period of study, as I once learned to my infinite inconvenience, always rigorously insisted upon.

Let us distinguish, then, between reform and improvement. We can all improve, let us hope that we do not all stand in need of reform. The word becomes offensive and impertinent when used too freely. It has the effect of the crack of a whip in a pasture full of quiet and contented animals. The peaceful creature who was solely intent upon his thistle, looks up and gives loud utterance to his impressions, and the unshod colts throw up their foolish heels into the air as if there were to be an end of all slow coaches from the date of their superfluous gambols."

If a feeling resulting from "the institution of *inferior medical schools* situated in wrong places," &c., originated the "great national project" of the Convention, it is truly surprising that the proceedings of the Convention do not contain even an allusion to the subject. Strange that no action was had upon it, and that no member of the convention even proposed it as a matter for deliberation! We concur, however, in the propriety of characterizing the objects of the Convention as objects of *improvement* rather than *reform*, but, with all our predilections for our *alma mater*, we are at a loss to perceive the propriety of assuming that the distinction applies *par excellence* to the Boston School. We have contended, on a previous occasion, that as applicable to medical Institutions, and the medical profession of the country generally, the terms improvement, and progress, are more appropriate than reform. It were easy to show from the history of both a continued career of advancement—neither have receded in excellence and usefulness, but the course of each has been steadily onward—how, then, can they be said to need reform. They are both capable of being bettered, and to combine and concentrate efforts for this end, we presume, was the prime purpose for which the convention was projected and carried into effect. In pursuance of this end the Convention adopted resolutions, recommending certain improvements in the system of instruction at medical schools. Now in these recommended improvements had the Boston School anticipated the action of the Convention so as to render this action inapplicable to it?

Dr, H. says: "In this University there was a full complement of instructors." Would it not have been more strictly correct to say there *is* a full complement, for, if we mistake not, the number recommended by the Convention, viz., seven, did not exist at the time the first Convention was held? It was then but six, anatomy and operative surgery being taught by the same professor, It has since been increased to eight. It was recommended by the Convention that dissections be rendered obligatory upon pupils before graduation, yet, we find by the reply to the interrogatory whether at that school the student is required to dissect, that it is voluntary with the student. Some other Institutions have already adopted this improvement. Again, the Convention recommended that Medical schools take measures to insure constant attendance of pupils at lectures. Is this done at the Boston School? And, again, as respects the still more important improvement of extending the lecture term to six months, we have yet to be informed that our venerated *alma mater* has followed the example of other schools in acceding to this improvement. We shall surely not be accused of a desire to depreciate that institution of all others, but we must say we are at a loss to perceive that it differs from the majority of the *well located* medical institutions of our country so far as to be excepted from any application of the recommendations of the Convention.

In the quotation which follows, the writer reiterates the stereotyped complaint of the multiplication of medical schools, and the pernicious effect of competition. The reader will, of course, form his own opinion of the taste evinced in the allusions to other teachers, which is indulged in an introductory lecture by a newly appointed professor. As respects the effects of competition in medical schools we can perceive no reason why it should not have its advantages here, as in every other sphere of effort. We know it is customary to attribute most of the evils under which the profession labors to this source, but we believe no reason can be assigned for the opinion except that it has become habitual. We cannot devote space, in this connection, to the discussion of the topic, but we would simply ask two or three questions, to direct the attention of our readers to facts which settle the matter practically and fully: Is the University of Pennsylvania less deservedly distinguished in its educational advantages than it probably would have been, had the Jefferson school never been organized? Has the Jefferson school suffered by competition with the several other institutions which have been more recently established in Philadelphia? What was the condition of the College of Physicians and Surgeons of the city of New-York before the University school went into operation and what is its comparative reputation now? If our Boston friends will reflect upon these questions, we honestly believe they would not be alarmed at the prospect of a rival medical school in their own city. Excellent as the school now in existence there is, it would become, in our opinion, better, and more flourishing under the stimulus of competition. We state this as a prediction, for the time is probably not far distant when the experiment will be practically tried. But to the quotation which has suggested these desultory remarks:—

"The extreme and rapid multiplication of medical schools has certainly had its use in educating the teachers, if not the students, and perhaps in giving a little training to some of those who might have gone into practice without any. I need not say what bad consequences it has had; I have hinted at them already, and the country is full of loud complaints on the subject. So long as the moderate compensation of the teacher is an inducement to unemployed young men; so long as the poor title, bestowed upon every itinerant who juggles a living out of the pseudo-sciences, can tickle the ears of half-taught practitioners, will legislatures be teased to grant charters for new schools, not only uncalled for by any public want, but tending directly to lower the standard of education. Competition is a good thing in its place, but it has blown up hundreds of poor wretches who trusted themselves in *Western* steamboats; and if suffered to run riot through our medical institutions they will be true to the parallel of hot fires, thin boilers, close valves and their inevitable consequences. There is a natural fitness of circumstances that might be regarded with some advantage. Men do not establish factories on hill-tops, where the water that turns the wheels must all be pumped up by hand. They do not organize infant schools in diving bells, where the light is to be let in through a bull's eye, and the air to be sent down in demijohns. Rivalry in medical institutions must always exist, but this business of underbidding and under-feeding, this farming out of medical students, like town paupers, to the lowest contractor, must eventually be arrested. If the law cannot do it by the necessary discrimination, organized public opinion can and will do it. And the time must come when those institutions which cannot by any possibility afford practical instruction in the most important branches of the profession will cease to be recognised as capable of giving a full title to public confidence. It is but the addition of three or four letters to those which designate the medical graduate, and the *Doctor Medicinæ Pennsylvaniensis* or *Harvardiensis* is as well known as the Parisian graduate by the title which he never fails to claim, and the equality which now confounds the most important differences is at once overthrown and abolished!"

Our readers have not failed to observe from the tenor of a note annexed to the extract preceding the last, that Dr. Holmes entertains a very low opinion of Western Schools. His ideas of Western and Southern teachers may be gathered from the extract which will follow. His remarks upon multiplied schools, and "inferior medical schools located in wrong places," "unemployed young men becoming teachers for the moderate compensation," &c., seems to have reference especially to the West and South. We cannot let this pass without a remark or two, although our western and southern brethren are abundantly able to defend themselves, and perhaps will not thank us for volunteering any comments on this attack, for so we must term it. Our remarks will be rather apologetic for the writer than otherwise. We have some little practical acquaintance with "Boston notions." One of these notions is, that Boston is the *ne plus ultra* of all places, another is, that it is the *sine qua non.* We should be sorry to seem obnoxious to the accusation of being wanting in respect for the metropolis of our native state. The accusation would be groundless, We delight to honor it as the seat of learning, science and refinement; but it does not absorb all the talent, acquirement, and intellectual resources of the country.

It is really amusing to a visitor at New England to observe how little all that that pertains to the "great states of the distant West" (to quote the writer's expression) is appreciated. The Bostonian thinks it passing strange that in Europe his white skin occasions surprise. The ideas which prevail in New England respecting the West, appear not less strange to the citizen of the latter. The author of the address before us, in the remarks to which we have directed the reader's attention, only expresses a common sectional sentiment. We do him the justice to believe that he supposes his remarks to be such palpable truisms, that no offence can possibly be taken, for, we presume he would not knowingly violate the rules of courtesy and good-breeding, to say nothing of medical ethics. That he honestly thinks the medical institutions of the distant West are "miserable apologies for medical schools" we have no doubt, and, furthermore, it is to his mind so obvious that it must be so, as to require no reserve in uttering the opinion. He could not probably be induced to speak disparingly of the schools which are, comparatively, in his neighborhood, viz., at Pittsfield, Woodstock, Castleton, Hanover, Bowdoin, all located in small villages. These are in New England, and cannot be charged with being "inferior schools wrongly located," the chairs filled with "unemployed young men," &c. New England needs them all. But in the "great states of the distant West" and South, what are the schools referred to? They are as follows:—One at Chicago, Illinois, a town now larger than was Boston when the Boston Medical School was instituted; one at La Porte, in the great state of Indiana; two at St. Louis, a city larger than was Boston twenty years ago; one at Cincinnati, one at Columbus, and one at Cleveland, these from the great state of Ohio; two in Kentucky, one at Lexington the other at Louisville; one in Tennesee; one in Louisana, at New Orleans; one at Charleston, South Carolina. These are the Medical Institutions of the distant West and South. We beg the reader to cast his eye upon the map of the United States, and compare the territory over which these schools are scattered, with the area of New England with its seven Medical Colleges, (five of which are in villages,) and more in contemplation!

We will not enter upon the delicate undertaking of instituting comparisons of individuals, but there can be no invidiousness in enumerating some of the teachers connected with the "miserable apologies for schools" in the distant West and South. The names of Drake, Caldwell, Gross, Dudley, Bartlett, Brainard, Linton, Blake, Delamater, Mussey, Lawson, Carpenter, Ackley, Dickson, Moultrie, Harrison, and others, are certainly familiar, not only at the South and West, but with the Medical Profession of the whole country.

We must waive farther remark, and after presenting the extract already referred to, proceed to notice some Introductory lectures emanating from Western Schools, of which we have several on file.

"There are many peculiarities in the medical character of this section of the country. Our position in New England, a little out of the broad current, our distinct origin, our hereditary habits, manifest their their influence in the shades of professional as well as political character. We may expect to find the New Englander as cool, as shrewd, as practical in medicine as in business. But his peculiarities are best displayed in the medical teacher and the medical public. The first is singularly calm, simple and didactic, as compared with many of his distant brethren; the second cautious, sedate, respectful to a degree which the fiery children of the south would call tame and submissive. When the annual flowering of "Introductory Lectures" takes place, it may be seen that the colors are generally higher as the distance from equator is less, and that the gayest display is from those that have had the advantage of the last rays of the setting sun; the efflorescence of scientific enthusiasm on the banks of the Mississippi or Missouri. Whether it be the coldness of Northern winds or the sterility of Eastern soil, there is less leaving out in proportion to the yield, and that of a less glaring aspect in our sober nurseries of knowledge than in those of our Southern and Western friends. Our danger is in the direction of *sensible dulness*, and theirs in that of glittering wordiness."

*An Introductory address delivered in the Medical College of Ohio, Nov. 2d* 1847. By L. M. Lawson, M. D. prof. of Materia Medica and general Pathology in the Medical College of Ohio.

This is the title of the publication which comes next in order. Dr. Lawson has lately become connected with the school at Cincinnati, having formerly been a member of the Faculty of the College at Lexington. He is editor of the Western Lancet, a popular and able Medical Journal.

Having defined the philosophical character of the several elements which combine to constitute medicine as a science and art, the lecturer sketches briefly the history of medicine from the votive tablets of the Æsculapian temples, downward to the 19th century; he next considers the sense in which our science is imperfect, and comments on certain errors of medical philosophising, especially the error of trusting to naked facts, and the absurdity of supposing that statistics are adequate to develope principles of pathology and therapeutics applicable to all individual cases. We give an extract relating to the latter topic:

"This so-called science of fact and experience, even when narrowed down to what is termed mathematical probabilities, is, it seems to me, after all a mere science of casualty. Suppose, for illustration, we have a box containing four balls of two different colors, white and black,—but we do not known how many white nor how many black. To determine this point, I draw four times; (putting back the ball each time) and the result is, that I have drawn *three* white balls, and *one* black one. I now base upon this *experiment* three hypotheses: 1. There may be *three* white balls and *one* black. 2. There may be *two* white and *two* black. Or, 3. *One* white and *three* black. One of these hypotheses must of necessity be true.

Now these hypotheses place us in possession of certain numerators, which represent the probabilities of the truth of each hypotheses, and upon careful calculation the following is the result: Upon the first hypotheses, the chance of drawing a white ball is $\frac{3}{4}$; upon the *second*, $\frac{1}{2}$; upon the third, $\frac{1}{4}$, the sum of which is $\frac{116}{184}$. And of course, reversed, we find the chance of drawing a black ball upon these three hypotheses, respectively, $\frac{1}{4}$, $\frac{2}{4}$, $\frac{3}{4}$,—the sum being $\frac{68}{184}$; and the sum of the two probabilities is 1.

Now based upon this *experience*—for it is all experience without any principle—I stake my life upon the chance of drawing a white ball at the next trial—which would be the case in medical practice based alone upon *experience*—I draw the ball, when, lo! it is *black!*

This doctrine of *experience*, as developed in mathematical probabilities, is fully applicable to Louis' numerical method of arriving at great laws, and also to experience without principles in all of its forms. It will be seen that in the above illustration, the *relative* probability really existed, but in my own attempt to make a practical application it failed; and not the slightest doubt can remain that such rules of action, when applied to the practice of medicine, will fail more frequently than succeed. Hence, I repeat, the practice based upon sach rules is nothing more, after all, than a science of casualty.

And now, gentlemen, if there be truth in history or reason in man—if our lives are not fables and our philosophy a caricature and a lie—then we have a science based upon facts and clothed in the habiliments of truth and reason; a science which has been handed down from age to age, and from nation to nation, until now its broad sheen illumines the world."

The lecture concludes with an animated appeal to the class in behalf of diligence, perseverance, and elevated aims. The address is creditable as a literary effort, catholic in its spirit, and altogether a good introductory.

The Medical College of Ohio furnishes another Introductory, which is from the pen of Dr. John P. Harrison, Professor of the Theory and Practice of Medicine in that Institution, the subject being the "*State and Prospects of the Medical Profession.*" It might be characterized as a defense of the Medical Profession from attacks by its own members. The writer repudiates and disproves sentiments so freely enunciated of late, which impute to the Profession, as a body, ignorance and degradation—a sentiment not emanating from the Public, but, *proh pudor!* from the Profession itself. He is far from thinking that the present state of the profession merits contempt, and he thinks that its prospects should inspire us with animation and zeal. We are glad to see such views from one whose opinions should command attention and respect.

We select as a specimen of the performance the writer's remarks on the multiplication of medical schools, by which it will be perceived that sentiments upon this topic expressed by the Professor whose discourse was first noticed in the present article, are by no means so universally entertained, that a writer is authorized to assume them as established truths, in which light the Professor alluded to presents them. We commend the extract to the reader's attentive perusal:—

The Medical Schools of the United States have increased very rapidly within a few years. Some excellent men look upon this rapid augmentation of Medical Schools as fraught with disastrous consequences to the highest interests of the profession. We cannot agree in this ominous foreboding—in this conscientious alarm at the quick process of their multiplication.

On the contrary, present good redounds from this source, and great future advantages will flow from their establishment. Four present benefits arise from the creation of Schools of Medicine in various parts of our country. In the first place, regular and properly organized schools of medicine repress quackery. By a regular Medical School we mean one that professes to teach the science and art of curing diseases, as the principles of the science are understood, and the measures of the art are employed, among all christian, civilized, and refined people. And by a properly organized School is intended one which, in a proper location, has the common number of Chairs, teaching all the important branches, and whose requisitions for graduation are the same as obtained in all respectable institutions.

It is objected to the multiplication of Medical Schools, that they furnish such facilities and hold out such inducements, as to tempt young men, with insufficient preparation, to enter upon the study of Medicine, and that by granting diplomas upon inadequate proofs of acquirements in medicine, they degrade the whole profession. The objector has never, perhaps, reflected, because he may not be familiar with the real condition of things, that were there no regular medical schools, irregular practitioners would increase more rapidly than they do at present, and that it is best to throw in the market a genuine article, of even inferior value, than to allow an altogether spurious one to take its place. As to the temptation given young men to induce them to solicit the doctorate, which, it is contended, our Medical Schools are derelict in, one answer is sufficient to show the fallacy of the objection. Responsibility, however slight in its exactions, is preferable to irresponsibility in any shape or degree. Suppose there were but four or five Medical Schools in our country, instead of thirty-seven, the actual number at present. With the rapidly increasing population of the United States, it is apparent that these five or six, or even twice five or six, would be totally inadequate to supply annually the proper number of physicians for our country.

But there is another advantage derived from the multiplication of medical schools entirely pretermitted by our objector. In this matter, as in all other departments of human life, a gradation will be observed. The older, or more celebrated institutions will attract the largest number of students ; even some of the students who attend one course at the less celebrated schools, will resort at their second session of lectures, to the more renowned seats of instruction. Thus, whilst the less established schools are patronised to a sufficient extent to keep them afloat on the tide, those of more celebrity will maintain their 'high and palmy state."

Again, the number of our schools of medicine has already awakened, and will keep alive, a generous spirit of emulation. This emulous, and stirring spirit of competition provokes to greater exertion in teaching, leads to a more careful selection of professors, and eminently conduces to a more liberal collection of apparatus, and of museums, by which the lectures delivered shall be illustrated and enforced. Already are these beneficial results witnessed, and with the march of time they will go on to develope their force and brilliancy with accumulated activity. This generous spirit of emulation has very noticeably promoted medical literature in our country. This encouraging sign of the times we shall more particularly notice.

A still additional auspicious influence exercised by our medical schools is the direct bearing they have in harmonizing the profession in all its high aims, and ulterior designs of improvement. This effect, by a natural and very operative principle, will follow from similarity of pursuit, and an ardent breathing after the same grand and glorious ends. If our medical schools are thus united in the holy brotherhood of science and humanity, they will send down, through all ranks of the profession, the same noble aspirations by which they are animated, in reaching after higher manifestations of excellence, and in maintaining a liberal union with each other in all the great purposes of their endowment.

The future or more remote results of this multiplication of schools of medical education, will be realized by young aspirants, who may hereafter devote themselves to the study of medicine. A hope, an aspiration, a determination, to become a teacher of medicine, will actuate many young men, and impel them to a more thorough preliminary education, as well as to a more protracted course of medical instruction. Open wide the door of advancement; present the prizes of honor and reputation, and emolument, to our young medical aspirants, and call up talent and industry, and ambition, from various portions of the land; thus genius will emerge from its obscurity, and deck itself with trophies won from nature's mysteries, and subjecting the contributions of science to the accomplishment of the ultimate objects of all professional efforts—the happiness of our race, the profession will arise superior to the petty assaults of its detractors, and vindicate its character from all the aspersions of ignorance,"

Passing, now, from the metropolis, *de facto*, to the metropolis, *de jure*, of the noble state of Ohio, we have from the Willoughby Medical College, recently transferred to Columbus from the village of Willoughby, an introductory by Prof. Butterfield. As the present course, to which Prof. Butterfield's address was a public introductory, is the first course of lectures in the new location of the school, the lecturer devotes a considerable portion of the discourse to considerations which this fact appropriately suggested. He appeals to the community of Columbus for encouragement and support in the enterprise of establishing a Medical school in that city. It would seem that the appeal had not been in vain, for, since the commencement of the session, a wealthy and liberal citizen has donated thirty thousand dollars for founding and sustaining a Medical College in that place, an act of munificent liberality for such an object, which is without a parallel in this country. The school is to be reorganized under a new charter, and promises, with the efforts of an able faculty, to become one of the most flourishing of the institutions of the west.

It has been intimated that it is to be named after the benefactor, the Starling Medical College. We trust that this example of judicious liberality may not be without its happy influence in other places, and that some of those who combine wealth and generosity will be incited by this precedent to give their bounty this direction. How can they serve better the cause of science and humanity! In what other department of public enterprise, and philanthropic effort, can a greater amount of good be effected by a moderate endowment! We quote the peroration of Dr. Butterfield's lecture:

"There is much in it [the medical profession] to command our love and respect. It embraces one of the highest, most benevolent and useful of human pursuits. Noble examples by hundreds, living and dead, are before you, to stimulate your industry and excite your ambition. I hope and trust there are many before me, whose footsteps upon the sands of time shall remain so long, that the *stone* shall perpetuate their momorial for ever.

I might dwell somewhat upon the self-devoted heroism, which disregards dangers, before which the stoutest warriors quail, and which constitutes a necessary element in the training and practice of every true physician ; of the scenes of suffering and wretchedness of which he is the daily witness, and through which he moves as the ministering angel ; but time forbids,

I see the noble McWilliams, steering with his own hands, the ill-fated steamer, through the death-dealing fens of the Niger, while all, officers and men, save one, and he at the engine, are dead or dlsabled. I read the record of another death, the fourth physician who has, during the present epidemic in New Orleans, fallen. I read almost daily of one, and another, and another, in New York, in Boston, in Baltimore, in Montreal, in Quebec, who have died victims to the faithful discharge of their duty. I see always, when the pestilence is walking in darkness and mowing down its hundreds and its thousands, the physician, calm and unappalled, at his post, battling with all his might, a far more irresistible foe than any mere human enemy ; and I am proud to be an humble votary of a profession which has offered up, and is still ready to offer up, its most costly and cherished jewels, as sacrifices upon the altar of the public good.

May you become, Gentlemen, worthy of the truly exaltedPro-fsesion towards which your efforts and ambition are directed."

If we were to indulge a sweeping criticism upon Introductory addresses, regarded as literary productions, we should say that they are generally rhetorically overstrained. It would seem that the Lecturer feels bound, at all hazards, to produce on that occasion a specimen of fine writing, and, hence, attempting the most difficult species of composition, an ornate, flowery style, as might be expected the production in some instances would become better the college sophomore, than the incumbent of a professorial chair. Our Boston friend would perhaps say this criticism applies to Western and Southern Lecturers more especially, but, if we do not err, it is not necessary to go out of Philadelphia, New York, or even New England for illustrations. It is fair to suppose, that, in some instances, the lavish profusion of tropes and metaphors, are due to the fact that the addresses were really written only to be pronounced before a medical class, and the writer, without affectation, is beguiled into the indiscretion of submitting his production to the calm perusal of those whose judgment and taste, if not cultivated, have been chastened by longer reflection, and the sober experiences of life. We have been led into this train of remark on examination of the two pamphlets before us remaining to be noticed. The first is by Dr. A. B. Shipman, Professor of Surgery in the Indiana Medical College. The second is by Dr. Daniel Drake, of Louisville, Ky.

Dr. Shipman's address is of the genus of florid introductories. We do not speak of it, however, as such, because it is *par excellence* a specimen of that genus. Perhaps some of the productions already noticed, are equally obnoxious to the criticism. This should be said, lest the prefatory remarks just made, may be thought to be designed for this particular application. The remarks were made in the present connection because they happened to be suggested, not because of their exclusive pertinency. We select the following extract:—

"A field of unexplored science lies before you. Enter upon it and make new discoveries. The Nervous System, where the Physiologist and Pathologist have discovered so much that is new and interesting, within a few years, is a field where future discoverers have much to hope for. The manner in which mind is united to matter, remains yet to be discovered. The diseased manifestations of mind, or the diseased tissues through which it is displayed, for we believe that the human mind is always bright and perfect, the diseased medium gives its apparent imperfections. We place a bright light in a clear and transparent glass,, and it sheds its clear and dazzling rays upon every object. We place it in a colored medium and we find it tinges every thing with its altered color, or we surround it with darkness and its light is gone; still it blazes clear as ever. The poor benighted idiot has still the same divine spark within, but it is surrounded and bedimmed with a dark, impenetrable, night. The various multitude of insane, have the soul shining out in a great variety of fantastic forms and hues, now flashing with the most intense and brilliant light, now flickering and wavering as if agitated by the wind, or going out in gloom and darkness. Here there is a field where new discoveries are to be sought. Some future Philosopher may yet arise to systematize and arrange these apparent mysteries, and clear away the mist that has hung like an impenetrable veil between the boundaries of mind and matter.

What a curious and wonderful organ is the brain! A tissue of matter, whose appearance gives little import of its high and lofty uses. Take, for example, the brain of Napoleon Bonaparte—look on that few ounces of soft pulpy matter, enclosed in its bony case, surrounded by membranes and pierced by nerves and blood vessels. That was the field whereon were marshalled the five hundred thousand troops, scattered over a great extent of country, yet so disciplined as to concentrate within an hour of each other on some field of strife. Each man had a place on that wonderful brain. The five hundred battles were fought there. The thirty millions of the French nation had a place; the snowy Alps; the Sacred Mountains of Palestine; Egypt; the Pyramids; the Nile; the Lybian Desert; the mighty Oriential Empire; all these, with vast plains for national glory, found a place; and yet there was room for his beloved Empress and darling boy.

The nervous system has much unexplored ground, that awaits seme future discoverer. Here, too, the structure gives little evidence of its astonishing and varied functions; a few small white cords, emerging from the brain and spinal marrow, acted on by distant objects, gives all the exquisite beauties of nature, in all her gorgeous coloring. Or the vibrations of the atmosphere all the harmonious sounds of the Universe.

The study of the organs, concerned in the manifestation of Mind, is one of vast and absorbing interest. Within a few years past, the physiology of the brain and nerves has been investigated with great zeal and ability. The researches of Gall, Spurzheim, Combe, and many others, have given an impulse to this department, that has cleared up many unexplained phenomena in physiological and metaphysical science. We do not pretend to endorse every thing that is claimed by these men; yet there are facts enough elicited in their investigations, to render their labors exceedingly valuable. It will not appear strange that great error may have been mixed up and blended with truth; especially, where the subject is surrounded with great obscurity. The ardent and sanguine, in their search for facts to sustain a favorite theory, may have seized hold of some plausible appearances, and baptized them into their creed. It should be your aim in all these vexed questions, to steer clear, on the one hand, of an overweening credulity, or an obstinate scepticism. You must remember that absolute demonstration is not to be expected in matters appertaining to the science of mind. Your evidence must be inferential in many instances; and let me tell you, it is exceedingly unreasonable to doubt all that does not appear plausible. The human mind, after all, is one of the most incomprehensible and mysterious things in nature. Can any one explain its most simple act? Can you tell me how I raise my hand and place it on my breast? What connection has my will with the muscles of my arm? What sympathy has my eye with that distant grove, that lies like an island on the prairie? Or can you explain how, by an effort of the mind, we are transported to our distant firesides, see the familiar faces of our relatives and friends, as we left them a few weeks since; or how, with the velocity of thought, send our fancy to distant lands, skimming the boundless seas, scaling lofty mountains, sinking into sunless abysses, traversing the whole earth, and even up to the highest heaven, at the very foot of the throne of the Eternal.

What a mysterious thing is the human mind? the tenant of a home so perishable as the human body. A structure of bone and muscle, of membranes, nerves and blood vessels, liable to a thousand accidents, more perishable than idols made of wood and stone, yet the tenement of immortality.

Our business the ensuing winter will be in endeavoring to render this structure a place of comfort and security. Where shall we commence our labors? How so likely to succeed as to make ourselves thorough masters of this curious habitation, to keep it from decay, to prevent the worm and the insect from committing ravages, protecting it from the storms that sweep over the plains, and the mouldering damps that collect beneath its foundations, to guard against the devouring fire from within, and the scathing lightnings from without, the rude attack of hostile foes, or the secret ambush of hidden enemies. Like skillful engineers, our energies should be directed in warding off the attacks of the sappers and miners of life. Then, after having perfected yourselves in every branch of our art, you will go forth into the world on your errand of mercy."

Dr. Drake's address, which, as regards the topics handled, and the mode of handling them, differs from the usual character of introductories, is thus entitled:—*Strictures on some of the Defects and Infirmities of Intellectual and Moral Character, in Students of Medicine.*"

The subject is novel, and would hardly be selected for an *ad captandum* effect upon the auditory before which it was pronounced. The writer is a veteran teacher, and has had abundant opportunities to study the moral and intellectual defects of medical students. He rehearses the fruits of his observations with great plainness of speech—there is no disposition to mince matters either in language or ideas. The lecture was written to communicate wholesome truths, not to tickle the ears of his hearers by that homage of adulation to which custom, to some extent, has conferred a claim. Prof. D. has no need to concede aught to the validity of such a claim. Our readers will desire to know what are the prominent defects and infirmities upon which the writer comments. He divides Medical students into eleven groups. We must content ourselves with the bare enumeration of the titles of each group. We could not do justice to his remarks upon them severally, without transferring the entire discourse to our columns.

The *first* group, consists of the *best* students—the best in a moral and intellectual sense. The *second* group, comprehends students who, for the sake of becoming fully qualified for the duties of professional life, are attending their third course of lectures. The *third* group, is composed of gentlemen somewhat advanced in life, who have entered on the practical duties of the profession before graduating. The *fourth* group, consists of those who have commenced the study of medicine without preparation. A *fifth* group, is made up of those who feel themselves under the necessity of going into practice after attending a single course of lectures. The members of the *sixth* group, are those who are altogether intent on *practical* matters. They who comprise the *seventh* group, are they who have unfortunately engaged in the study of medicine with inadequate capacities. An *eighth* group, consists of those who lack *punctuality*. Students who cultivate society during the session, compose a *ninth* group. The *tenth* group, embraces the dissipated. The *last* group, consists of "bullies and desperadoes." We trust, for the honor of Southern schools, that the last group includes but a small proportion of those who are assembled at their halls of instruction.

The reader will perceive from the foregoing classification, that numerous important topics are embraced in the scope of this address, and to say that the reader will find them ably treated, is needless, for who would expect otherwise from the well known ability of the author. The portion of the lecture which we shall select for quotation is the peroration, in which the talented lecturer displays that American spirit which we should be glad to see cherished and cultivated more than it is, within the rank of our profession:—

"In the fourth place, our students do not propose to themselves, that they will strive to enlarge the boundaries of our science. But why should they not? The American mind is not inferior in strength or invention to that of Europe. In the science of government—in jurisprudence—in eloquence—in war—in agriculture—in the fine and useful arts, it has shown itself quite equal, often superior, to the European mind. Why is it, then, that *we* continue to look, almost exclusively, to our medical brethren on the other side of the Ocean, and not to ourselves for discoveries and improvements? France, Germany, England, Scotland, and Ireland, are respectively cultivating the profession, as if the duty of enlarging its boundaries, rested on one only. The physicians of those several kingdoms, do not turn to each other with an eye of dependence, but of emulation. In this country, the reverse prevails. To acquire a tolerable knowledge of what is done abroad, too often constitutes the sum total of the ambition of the best minds among us, while hundreds do not even aspire to that. It is time that we threw off the shackles of colonial dependence, and assumed a higher position. Once, like callow nestlings, we of necessity swallowed whatever our European nursing mothers dropped into our gaping throats; but we are no longer unfledged, and should spread out our growing pinions, and begin a life of independent activity. This resolution once taken, success would not be doubtful, But who should take it? Those who are now in, or those who are aspiring to the profession? I answer, *all*, but especially the latter—you and your fellow students throughout the Union;—for the old are too inert, the middle aged too indifferent, and a majority of both classes, too little prepared by early discipline, or lofty aspiration, to enter on a career of glory. Our hopes, therefore, must rest on you more than your preceptors and seniors. Would to God, my young friends, that I could kindle in your hearts a flame of patriotic ambition, so intense as to burn up every germ of *servile* dependence on the physicians of the old world! Would that I could fill you imaginations with bright and beautiful visions of the future! But should

I do so, remember that the most glorious conceptions will not, of themselves, secure the objects which they embody. The conceptions of mind which tell on the progress of an individual, a class, or a nation, comprehend both causes and effects. The little child as eagerly puts forth its hands to pluck the flower which hangs beyond its reach, as that which blooms within its grasp. Experience tends to correct this constitutional error of infancy; but in many minds the correction is so slow and imperfect, that the man, in this trait of character, remains a child; and while he indulges in captivating visions of excellence, which cost no effort, neglects the labors which alone can convert them into living realities. They who do not rise above this infirmity of our nature, will do nothing towards emancipating us from our unequal dependence on Europe. They who surmount it, will contribute to the foundation of an American profession. To this great and brilliant enterprise your *alma mater* now calls you—every one, the humblest not less than the highest, for all may contribute something. How happy would she be, to see every heart here, and in all the sister schools of the Continent, quickened into life, and filled with a moral courage equal to the task! Then, the question with the young aspirant would no longer be—What saith the seer of other countries? but of our own, our beloved native land: then, improvement would roll onward, like a mighty river—deep and fertilizing as the Mississippi—transparent and uniform as the St. Lawrence! The fame of its waters would spread to other lands; and many of their young men would come and drink. American students as they traversed the ocean for the East, would then greet European students on their way to the West; and on reaching the schools of the old world, would feel no humiliation; but in the pride of a glowing patriotism, would be able to exclaim to those around them—"If we have sought the banks of the Thames and the Seine, yon young men are treading those of the Deleware, the Hudson and the Ohio."

We have now reached the end of the introductories which had accumulated up to the time of commencing this article. We then supposed that on committing the last of the budget to the hands of the Printer's Devil, our labors in this line would be finished. But, as the cant expression goes, "there are a few more of the same sort left," with which we have been favored quite recently. These we must reserve for another number.

